# A Self-Powered Wireless Water Quality Sensing Network Enabling Smart Monitoring of Biological and Chemical Stability in Supply Systems

**DOI:** 10.3390/s20041125

**Published:** 2020-02-19

**Authors:** Marco Carminati, Andrea Turolla, Lorenzo Mezzera, Michele Di Mauro, Marco Tizzoni, Gaia Pani, Francesco Zanetto, Jacopo Foschi, Manuela Antonelli

**Affiliations:** 1 Dipartimento di Elettronica, Informazione e Bioingegneria (DEIB), Politecnico di Milano, Milano 20133, Italy; 2 Department of Civil and Environmental Engineering (DICA)—Environmental Section, Politecnico di Milano, Milano 20133, Italy

**Keywords:** wireless sensor network, smart pipe, biofilm, scaling, impedance, interdigitated microelectrodes, energy harvesting

## Abstract

A smart, safe, and efficient management of water is fundamental for both developed and developing countries. Several wireless sensor networks have been proposed for real-time monitoring of drinking water quantity and quality, both in the environment and in pipelines. However, surface fouling significantly affects the long-term reliability of pipes and sensors installed in-line. To address this relevant issue, we presented a multi-parameter sensing node embedding a miniaturized slime monitor able to estimate the micrometric thickness and type of slime. The measurement of thin deposits in pipes is descriptive of water biological and chemical stability and enables early warning functions, predictive maintenance, and more efficient management processes. After the description of the sensing node, the related electronics, and the data processing strategies, we presented the results of a two-month validation in the field of a three-node pilot network. Furthermore, self-powering by means of direct energy harvesting from the water flowing through the sensing node was also demonstrated. The robustness and low cost of this solution enable its upscaling to larger monitoring networks, paving the way to water monitoring with unprecedented spatio-temporal resolution.

## 1. Introduction

Water is irrefutably a critical resource for mankind, with major sanitary and economical relevance. Water availability and quality directly affect human health, nutrition, agriculture, and the environment. Correct handling of the full cycle of water has an impact on marine ecosystems, hydrogeological safety, and it is pivotal within the paradigm of smart cities, especially in terms of sustainability. In fact, safer and more efficient management of water is fundamental for densely-inhabited metropolitan contexts, along with other distributed infrastructures, monitoring air pollution, traffic and mobility, waste management, etc.

Conventionally, water quality is monitored by periodic sampling and laboratory chemical analysis. This approach grants excellent sensitivity and selectivity in detection, at the price of limited spatio-temporal resolution [1]. In-line instruments, monitoring mostly electrochemical parameters, are being introduced as well in a few locations. The current trends of miniaturization and integration of functions enabled by the fast-paced development of consumer electronics and wireless technologies provide suitable infrastructures for pervasive sensorization of the environment. Several wireless sensor networks have been proposed to monitor water quality in real-time, both in urban [2] and remote scenarios [3], as well as in oceans [4,5,6], rivers [7,8,9], and water reservoirs [10,11]. Although several types of network topologies, hierarchies, radio protocols, and data elaboration approaches [12,13,14,15,16,17] have been proposed in the last decade, no consolidated solution has emerged so far. 

In this work, we focused on the drinking water supply infrastructure, and we addressed one of the main related issues: the biological and chemical stability of water in distribution systems [18,19]. These properties are of paramount importance for safe and reliable water supply. In fact, they must be guaranteed in order to avoid the occurrence of many related problems, as worsening of water quality resulting in microbiological risk [20], system failure due to pipe clogging [21], water loss due to leakages in pipes [22], or sensors malfunctioning due to fouling [23,24]. In this view, real-time monitoring of water biological and chemical stability could enable early warning functions, predictive maintenance, and more efficient management processes in the distribution system. We proposed a sensing node, combining conventional chemo-physical sensors with a novel impedance monitor of the surface fouling, able to estimate its thickness and discriminate its origin (biological vs. inorganic). It offers faster response and better sensitivity than low-cost optical approaches [25].

Along with above-cited problems, the increasing aging of the drinking water distribution infrastructure, in several cases one-century old, poses severe issues of safety and maintenance. For instance, in Italy, the fraction of water reaching domestic users dropped from 62.6% in 2012 to 58.6% in 2015 [26], meaning that the average leakage loss of the network exceeds 40%, and the trend is not improving. This situation produces side effects, such as a widespread and unmotivated suspicion towards the quality of drinking water (in Italy, 29% of families avoid drinking tap water, as reported by the national statistics institute [26]). Thus, one of the main motivations of this work, along with providing the network manager with real-time monitoring instruments and maps, is to invert this trend and improve the confidence of citizens towards the quality of tap water.

A key aspect of the pervasive diffusion of sensing nodes is their energetic autonomy. Water and energy are strictly entangled: energy is used to treat and distribute water, while energy can be also harvested from water flows, especially in order to monitor its quantity and quality [27]. A demonstration of this nexus is the flourishing of self-powered devices and networks for smart metering, leveraging Internet-of-Things (IoT) electronics for monitoring water quality and consumption, especially aiming at leakage reduction [28,29]. Energy can be harvested from the flow of fluids in several ways [30,31], improving the efficiency, stability, and compactness commonly achieved, for instance, by solar panels [8]. Here, we demonstrated the possibility to self-power the proposed sensing node by means of an energy harvesting unit that enables its installation in remote sites, off the grid.

Finally, it must be underlined that an additional challenge in this context is posed by a limited budget. This aspect has been already highlighted by other works [2,11]: clearly, a large-scale diffusion of water wireless sensing networks can only be achieved if the unitary cost of the nodes is affordable. A similar paradigm, in which the spatio-temporal resolution of environmental monitoring maps is significantly improved by the diffusion of low-cost measuring units and participatory sensing, based on emerging miniaturized technologies and complementary to the few (often fixed) governmental reference monitoring stations, has been observed in monitoring air quality [32], radioactivity [33,34], and local forecasting of rainfalls [35].

## 2. System Architecture

**Figure 1:**
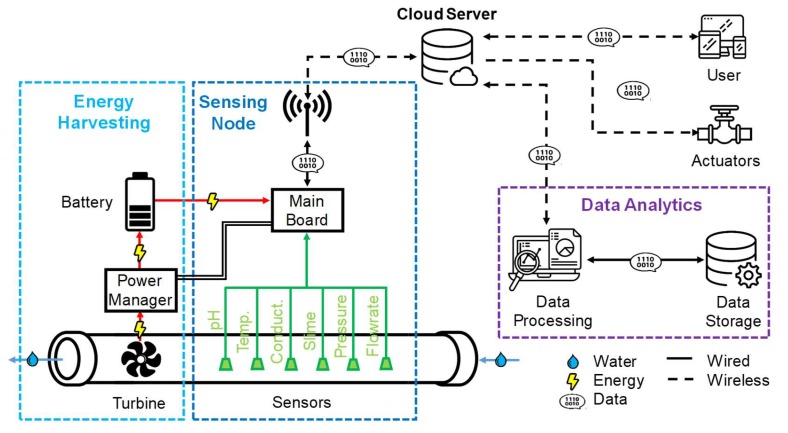
The architecture of the proposed network composed of sensing nodes (monitoring 6 chemo-physical water parameters) powered by kinetic energy harvesting and connected through long-range radio links to a Cloud server, providing visualization, feedback control, and analytics.

The architecture of the wireless sensor network that we realized is pictured in Figure 1. Each sensing node measures in real-time six parameters of the water flowing inside the distribution pipe: temperature, conductivity, pH, pressure, flow rate, and the thickness of slime depositing on the inner pipe surface. Additional sensors, in particular optical and electrochemical ones, measuring, for instance, transmittance, turbidity, dissolved oxygen, heavy metals, or residual chlorine can be easily added since the sensing node is endowed with auxiliary analog inputs and outputs. A microcontroller manages the operation of the sensing node and performs basic calibration and fault detection of sensors. Data are periodically acquired and transmitted by means of a radio link to a Cloud server. For this application, we selected the GSM/GPRS cellular network, given its ubiquitous coverage of the territory (98% of the Italian territory by Telecom Italia provider). Other low-power radio infrastructures oriented to IoT applications, such as a LoRaWAN and Sigfox, which are in the process of consolidation, can replace the GSM when they reach the same coverage, especially required for rural areas crossed by the water distribution network. Security is, of course, another key aspect to be taken into account when selecting the proper radio protocol.

**Figure 2:**
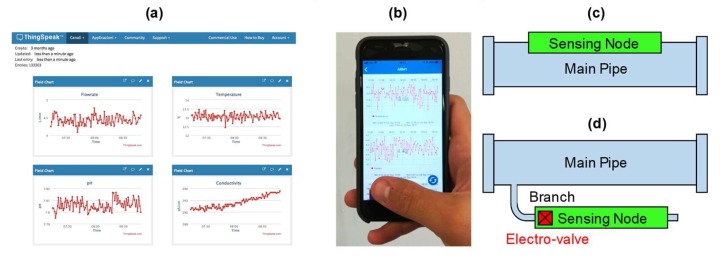
User interface of the ThingSpeak™ Cloud displaying in real-time acquired data on a password-protected website (**a**) and on smartphone app (**b**). Possible installation approaches of the sensing node: (**c**) in the main pipe or (**d**) in a derivation branch, whose flow can be controlled.

ThingSpeak™ (Mathworks) was selected as a Cloud server since it offers enough resources for this pilot demonstration in its free version. It enables to display data (both raw and processed) on a website (Figure 2a) as well as on portable devices through an app (Figure 2b). Data on the server can be: (i) sent to the user, typically the utility or agency managing the water distribution system, for continuous monitoring, (ii) used by automatic controllers to activate actuators in order to operate in closed-loop sections of the network and act, for instance, by closing electro-valves in response to sudden leakages or alterations of the water quality parameters, due to pipe degradation or pollution events, (iii) sent to a data analytics unit. Data can be processed by statistical tools in multiple ways and at several levels. They can be merged, correlated, and compared with additional heterogeneous data provided by other complementary sources. They can be used to initialize and fit models describing, for instance, the growth of bacteria or to train machine learning models to achieve predictive maintenance of the pipes and to optimize various treatment processes, often involving the injection of chemicals, such as disinfection by chlorine-based compounds.

The sensing node can be installed along the main pipeline (Figure 2c) or in a branch (Figure 2d), depending on installation and materials constraints. Since our prototypes have not been certified in terms of food compliance, they have been installed in branches for the pilot validation in the field. The unit also includes a digital signal to control an external electro-valve that can be open only during the measurement time, to minimize water loss. In our demonstration, a standard motorized ball electro-valve for ½” pipes (SEV3000 operated at +5 V DC and consuming 25 mWh in a full open-close cycle) has been employed.

The node can be powered by a +5 V DC power supply by a battery. It also comprises an energy harvesting system, extracting power from a turbine placed along the same pipe and, thus, enabling the energetic autonomy of the node. The turbine should be placed downstream of the sensors, in order to minimize the perturbation of the hydrodynamics in the measurement sites.

The choice of the sampling period is strictly related to the specific needs of the application and to the energy available for each node. The minimum sampling period is 15 s. The typical period used in the validation has been 1 min. In case real-time reactivity is not required, the sampling period can be increased to hours for routine monitoring, thus significantly reducing the average energy consumption, since the sensing node switches to sleep mode when not acquiring.

## 3. Sensors

### 3.1. Slime Sensor

**Figure 3:**
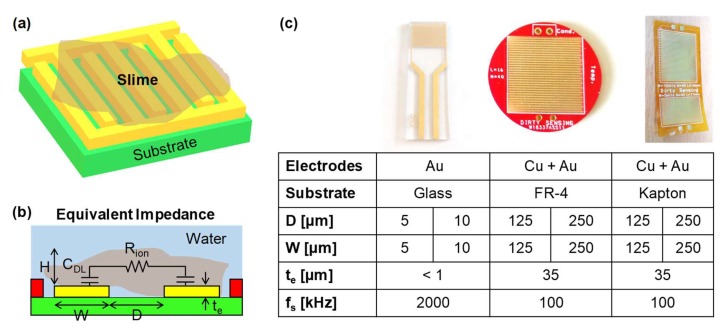
Slime thickness monitor based on planar interdigitated microelectrodes (**a**), of thickness t_e_, width W, and spacing D (**b**), fabricated in different technologies and geometries (**c**). Impedance is measured at a single frequency f_s_.

The deposition of a micrometric layer of slime on the surface of the pipe is measured by means of an impedance sensor. The sensor is based on closely-spaced microelectrodes, whose working principle is depicted in Figure 3. In detail, impedance is measured across two combs of interdigitated microelectrodes whose fingers (of width W and thickness t_e_) are separated by a spacing D. In order to maximize sensitivity, D should be matched with the thickness H of the deposit to be monitored. The equivalent small-signal impedance model (Figure 3b) is composed of the capacitance of the electrochemical double layer (C_DL_) in series to the ionic resistance (R_ion_), scaling with the liquid bulk conductivity (drinking water has usually a conductivity between 500 and 1000 μS/cm). No faradaic component is present since the voltage applied across the interface (50 mV) does not activate any redox reaction. The value of C_DL_ scales with the area of the electrodes, while D sets R_ion_ and the vertical sensitivity [36]. Given the coplanar geometry, conformal mapping expressions for a finite layer are used to estimate the value of R_ion_ [37]. In [38], we compared measured values of R_ion_ with the value provided by conformal mapping and finite-element simulations, finding a good agreement.

When a layer of slime deposits, it alters both C_DL_ and R_ion_. The displacement of ions due to the presence of an insulating body on the electrodes typically produces a decrease of C_DL_ and an increase of R_ion_. This technique is extensively used, for instance, to electrically monitor in-vitro the growth of colonies of biological cells [39]. Since the interfacial C_DL_ is extremely sensitive to surface events, at the sub-nanometer level, it is also very unstable over time; thus, we chose to monitor R_ion_. By measuring impedance at a frequency f_s_ larger than the corner frequency 1/(π·R_ion_·C_DL_), it is possible to shunt C_DL_ and directly probe R_ion_.

Experimental tests evidenced that the change of R_ion_, ΔR is linearly related to the thickness H of the slime, both in the case of biological (biofilm) and inorganic (limestone scaling) deposits. Interestingly, the slope of the response is the opposite: a decrease in the case of biological deposits (where the conductivity of the colony extracellular matrix is dominant) and an increase in case of inorganic deposits. After the proof-of-concept of the sensor [40], a significant amount of additional development has been carried out to improve the robustness and integration of the device under relevant operating conditions in the view of its application. Different materials and geometries have been systematically tested (Figure 3c). The commercial microelectrodes (DropSense) with D of 5 and 10 µm (fabricated by lithography) used in preliminary tests have been replaced with custom-designed electrodes produced with the conventional technology used for printed circuit boards (PCB). This manufacturing process has allowed a significant reduction in costs and more versatility in the designed geometries. These components are small, circular, gold-plated elements with electrical contacts on the back of the rigid FR-4 substrate. Moreover, the geometries of components have been defined so as to allow the housing at the inner surface of pipes with diameter down to 1”, and the spacing of microelectrodes has been set so as to target the expected deposit thickness H. Two different spacing values have been chosen, respectively of 125 and 250 µm, in the view of dealing with the characteristic thickness of slime in pipes.

Flexible electrodes, fabricated on Kapton substrates with the same geometries, have been also successfully realized (Figure 3c). If bending takes place along the longitudinal orientation of the metal fingers, the measured sensitivity is the same as of the rigid version [41]. Since they can be conformally applied inside ducts with bending radii below 1 cm, they represent a viable solution for pipes of small diameters, such as in industrial and domestic appliances. Their long-term stability in water has not yet been assessed, but the chemical robustness of Kapton allows to envision no issues.

The calibration of the sensor takes place in two phases. First, the cell constant, relating conductivity to the measured R_ion_ and affected by geometrical tolerances, is extracted once after fabrication for every single sensor. Three saline solutions at known conductivities are used for a simple linear calibration. Then, in order to obtain the value of the slime thickness H from R_ion_, a calibration response curve obtained in the laboratory, under controlled conditions, is used. Since R_ion_ is a function of the water salinity and temperature, and since the calibration curve is obtained at 25 °C for a specific conductivity (750 µS/cm), temperature and conductivity are simultaneously measured to correct the impedance value.

### 3.2. Other Chemo-Physical Sensors

The custom-developed slime sensor has been complemented by five standard sensors to create the multi-parameter sensing node. Temperature and pH are measured by a Sensorex S272CD probe. This is the most expensive sensor and is critical in terms of long-term stability. The choice of a commercial device is due to the higher reliability and precision with respect to non-consolidated miniaturized pH technologies present in the literature. The pH probe is a flat glass membrane suitable for in-flow measurements, embedding also a thermistor. The instrument resolution is higher than 0.1 °C between 0 and 50 °C and 0.1 between pH 0 and pH 14, respectively, for temperature and pH.

Conductivity is measured by a homemade low-cost miniaturized device. The development of a dedicated instrument has been carried out in order to improve the characteristics of existing commercial probes. Gold-coated connector pins are used for sensing, as described in [42]. Fault detection of sensors can be achieved by physical redundancy or model-based approaches (observers, such as a Kalman Filter used in avionics [43]). In order to increase the self-diagnostic capacity of the system, multiple identical conductivity sensors can be inserted. The geometry of the electrodes has been chosen to obtain an f_s_ below 100 kHz, allowing the use of a single-chip impedance detector (AD5933 by Analog Devices), to save space and complexity. The electrodes are spaced by a few millimeters and are sensitive to the passage of mm-sized impurities and micro-bubble. In the initial prototype, a vibrating linear motor has been inserted, allowing periodic shaking of the pins to remove micro-bubbles from the electrode surface, whose detrimental presence on time tracking of conductivity has been observed in the field tests in open tanks [44]. In the final prototype, this feature has been removed since (i) the presence of bubbles is very limited in closed and pressurized pipes, (ii) an algorithm for discarding artifacts based on low-pass filtering and combining the signals from multiple probes has been introduced. The resolution of the conductivity probe is better than 20 μS/cm between 50 and 2500 μS/cm, its intrinsic response time is sub-ms, and the minimum sampling period is 10 ms. 

The flow rate is measured by a commercial device (FL-808 by Digiten), providing a signal, which is frequency-modulated using a Hall effect sensor. The resolution is better than 0.1 L/s between 2.5 and 80 L/s. The pressure is measured by a commercial device (3525VG1/40-1.2 by Elco), providing an analog voltage signal for pressure in the range from 0 to 1.2 MPa. The resolution is 1.5% of the full-scale range.

### 3.3. Complete Sensing Node

**Figure 4:**
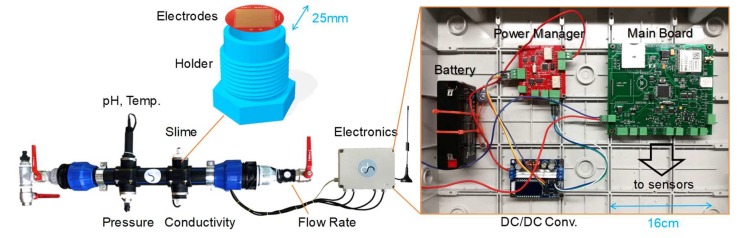
Photograph of the fully assembled node, showing the in-line sensors, the plastic holder of the slime monitor, the electronic boards, and the GSM antenna.

A picture of the fully assembled sensing node is visible in Figure 4. The placing of sensors is modular: they are located in sequence, along a 2′′ commercial HDPE pipe, commonly used for drinking water applications. As visible, four probes are inserted transversally to the water flow, screwed in holders for 1 ¼′′ pipes for fast disassembly and inspection (sealing is granted by Teflon tape). The design of the holder for the slime sensor is critical in terms of water tightness. The sensor PCB is glued on top of a plastic holder and sealed with resin. The adhesion of resin on the holder is crucial. The standard plastic material used for filament-based 3D printing (PLA) has proved to be not suitable for this application, and machined polypropylene has been used. The flow rate sensor, which can also be used as a harvesting turbine, is placed downstream. Shielded cables connect the sensors to the electronic unit, placed in a waterproof plastic box. The GSM antenna (ground-plane type) is connected through a coaxial cable and located above ground with a magnetic holder.

## 4. Electronics Design

**Figure 5:**
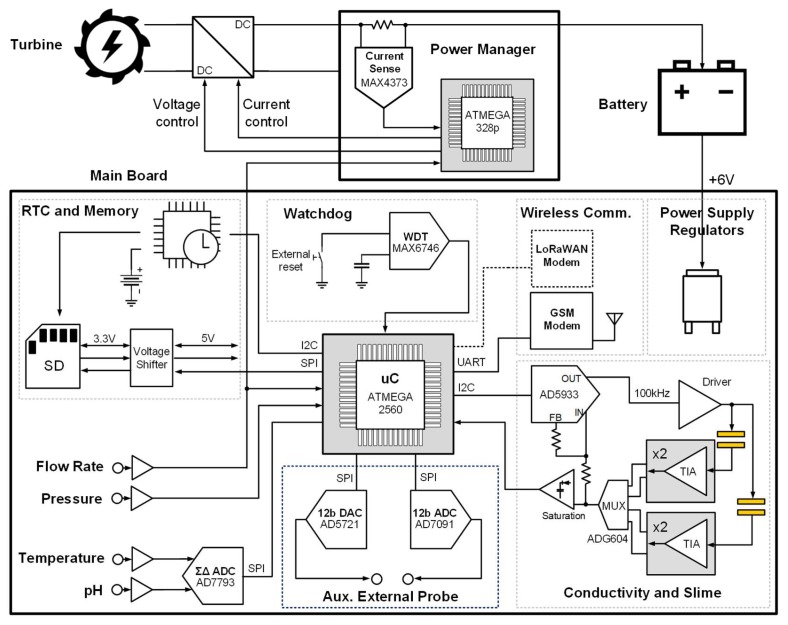
Scheme of the electronic platform, controlling the node: it is composed of the main board for conditioning, acquiring, and transmitting data and by a power management board for harvesting.

A custom acquisition and processing electronic platform was designed. The design of the electronics was led by the search for reliability and for the optimum balance between cost, simplicity, and compactness on one side and versatility and high-resolution performance on the other. In particular, special care was devoted to the design of the analog stages, which grants the accuracy (by properly choosing circuit topologies and low-tolerance components) and the resolution (i.e., low noise) of the measurements. Three generations of the electronic platform were developed during the project. The final system architecture is shown in Figure 5. The platform was composed of two units: the main unit performing conditioning, acquisition, processing, and transmission of signals from all the sensors and a separate power management unit taking care of the energy harvesting. Both units were based on 8-bit microcontrollers programmed in the Arduino development environment.

The main unit was controlled by an ATMega2560 microcontroller and interfaces with eight probes: pH, temperature, flow rate, pressure, two pairs of pin electrodes for conductivity, and two pairs of interdigitated planar microelectrode for slime. The signals from the pH probe (temperature, pH) were acquired by a 24-bit sigma-delta analog-to-digital converted (AD7793 by Analog Devices), setting a temperature-compensated reference and reading the value from the sensor, properly buffered at high impedance. The output of the flowmeter was frequency-modulated by the water speed, thanks to a Hall sensor, and it is read as a digital signal. The output of the manometer was an analog voltage (0–5 V), directly converted by the ADC embedded in the microcontroller, since its performances (resolution, linearity, sampling rate) were adequate. Impedance measurement was used for both the conductivity and the slime deposit. Impedance was measured by means of a single integrated circuit (AD5933 by Analog Devices) that simultaneously stimulated the electrodes with a sine wave (100 kHz) and performed an on-chip 12-bit FFT analysis with 1 ms time resolution. With a low-parasitics multiplexer, the microcontroller selected the correct channel and extracted the value of impedance, which was related to the physical dimensions of the electrodes. In a previous version [18], a more sophisticated analog demodulation solution (synchronous lock-in demodulation) was adopted to operate with f_s_ = 2 MHz due to microelectrodes’ dimensions. The choice to unify the circuit for conductivity and slime deposit greatly simplified the system with no performance degradation. Furthermore, to increase the versatility, an interface for an additional electrochemical probe was implemented. A 12-bit SPI DAC (AD5721 by Analog Devices) was coupled with a 12-bit single-channel ADC (AD7091 by Analog Devices).

Robustness to firmware faults (especially caused by issues in the GSM communication) was achieved by means of a watchdog on board (MAX6746, by Maxim). External watch-dog boards could be used for further robustness. Additionally, an SD memory and a real-time clock, providing time stamps, offered a memory buffer for data logging and in case of delays in the radio communication. 

Double wireless communication technology was implemented in the platform for long-range communication. A very robust GSM module (M10 by Quectel) performed a connection through the GSM (LTE) network. In addition, a low-power LoRaWAN module (mDot by Multitech) was included, requiring, in this case, the presence of the LoRa radio infrastructure. The interface between the main board and the modem was a standard serial digital bus, thus open to other types of protocols, such as Sigfox. The communication was mostly directed from the node toward the Cloud server. However, thanks to the “talk back” function of ThingSpeak™, it was possible to remotely set a few parameters of the board.

The main board was powered at +5 V, regulated by a filtered low-drop linear regulator. The battery chosen in the final prototype was a 6 V, 1200 mAh Sealed Lead-Acid battery (by RS Components). This type of battery is well consolidated and suited for IoT and industrial applications. Low cost, float charge for prolonged periods, and a large number of recharging cycles are three of the main advantages, especially for remote installation and long-term operation. A modular approach was chosen here, in order to adapt to different turbines and batteries (thus addressing different installation conditions). The energy harvesting unit was thus realized on a separate board, managed by a dedicated microcontroller (ATMega328p, an 8-bit very low-power and low-cost microcontroller). The battery management electronics was based on a DC/DC converter required since the input voltage coming from the turbine varied from 5 to 12 V, and the charging voltage switched from 2.4 V/cell (7.2 V in this case), during the charging phase, to 2.13 V/cell (6.4 V), during the float charge phase, when the battery was almost fully charged. A buck-boost DC/DC converter (by Haljia), switching at 180 kHz with adjustable output (5.5–30 V) and LCD display, was selected. A sensing resistor, coupled with a sense current amplifier (MAX4373 by Maxim), allowed the measurement of the value of the current absorbed by the battery, and the unit adaptively set the correct value of the charging voltage among the four current profiles (about 30, 45, 70, and 90 mA), using as input also the data from the flow rate probe. The physical realization of the compact boards is visible in Figure 4.

## 5. Experimental Results and Discussion

### 5.1. Laboratory Characterization of the Slime Monitor

**Figure 6:**
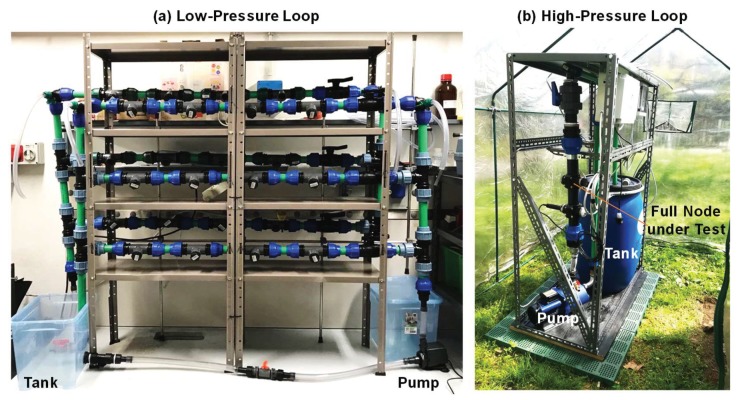
Laboratory setups for dynamic and continuous characterization of the sensors in the hydraulic loop with controlled conditions of temperature, flow, chemical concentration, and pressure: up to 4 bar in the low-pressure loop (**a**) and up to 9 bar in the high-pressure one (**b**).

In order to extend the readiness level of the slime monitor from the initial laboratory proof of feasibility [40] to a degree suitable for operation in the field, robustness had to be primarily addressed. For application in the field, the slime sensor (fabricated in a standard rigid PCB technology with D = W = 250 µm) was identified as the best option, given the expected thickness of deposits in the range of a fraction of mm. Its robustness was assessed during long-term measurement, and stress tests carried out in the laboratory under various conditions of temperature (up to 50 °C), pressure (up to 5 bar), and flow velocity on the surface of the sensor (up to 1.5 m/s) by a six-month continuous monitoring test performed with two hydraulic loops: a low-pressure (4 bar, Figure 6a) and a high-pressure (9 bar, Figure 6b) one. The former was used to characterize only slime sensors, while the latter for the validation of the node, fully equipped with all probes. Different and independent operating conditions could be determined in each channel of the low-pressure loop: flow velocity of 1, 3, and 5 cm/s and pressure from 0 to 4 bar. Water temperature could be controlled and reagents—to promote the formation or the cleaning-up of slime—were dosed in the tank reactor. Slime sensors were mounted on holders similar to the final ones (Figure 5) and could be easily inserted into dedicated sockets along the channels and aligned with the inner surface of pipes. Each channel could host up to four holders, in which polymeric or metallic element could be inserted in the place of sensors, to simulate the behavior of the inner surface of pipes of different materials. A total of 24 samples could be simultaneously subjected to controlled fouling.

**Figure 7:**
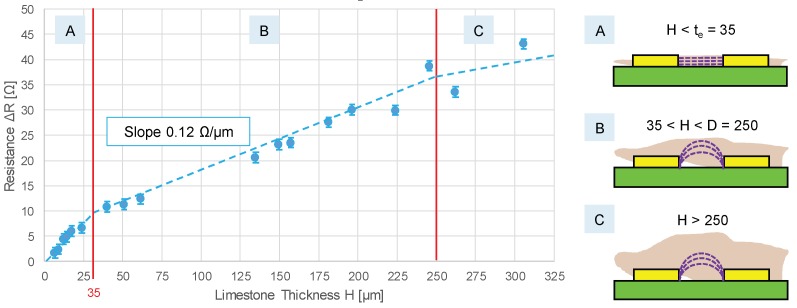
The response curve of the slime monitor for increasing thickness H of limestone, showing three regions of sensitivity.

As an example, the response curve of the sensor to inorganic scaling is reported in Figure 7. Here, a wide range of limestone thicknesses was explored, from a few microns to 300 µm, achieved by dosing the volume of a CaCO_3_-saturated solution of water whose evaporation was forced on the electrodes’ area. For thicknesses below 8 µm, an Atomic Force Microscope [40] was used to measure H, while for larger thicknesses, a gravimetric technique (combined with optical microscopy to estimate the fraction of covered area) was adopted.

As expected, three different regions (A–C) could be identified in the response curve. In the first region (A), when H was smaller than t_e_ (35 µm), slime only filled the gap between the electrodes, i.e., in the region where the electric field was the highest (electric field lines were horizontal and parallel as in a parallel-plate geometry). When H exceeded 35 µm (B), the layer of slime covered the electrodes, and the sensitivity was reduced to +0.12 Ω/µm. This number could be compared with +1.4 Ω/µm obtained with the same CaCO_3_ deposition achieved with microelectrodes on glass: in that case, sensitivity was an order of magnitude larger, thanks to the smaller D = 10 µm [40]. However, that sensitivity saturated for very thin layers above 10 µm. In region C, when H exceeded D (250 µm), the sensitivity was further reduced, since 95% of the electric field lines extended up to a thickness equal to D. Consequently, the recommended operating regions were A and B, confirming the optimal sizing in matching D with the maximum H to be measured. In the reference application of drinking water pipes, the typical values of H for scaling were in the order of hundreds of microns. Assuming a measurement accuracy better than 1 Ω, the resolution and accuracy in slime thickness estimation were better than 10 µm. This piece-wise linear and compressing response curve could be leveraged to accommodate a large dynamic range: for very thin H, a larger “gain” was provided by region A, while, for thicker H, the gain was reduced in region B. In other words, the monitor was typically operated in region B, but, for very thin layers, it could “zoom in” when H was in region A.

### 5.2. Field Validation of a Pilot Network

**Figure 8:**
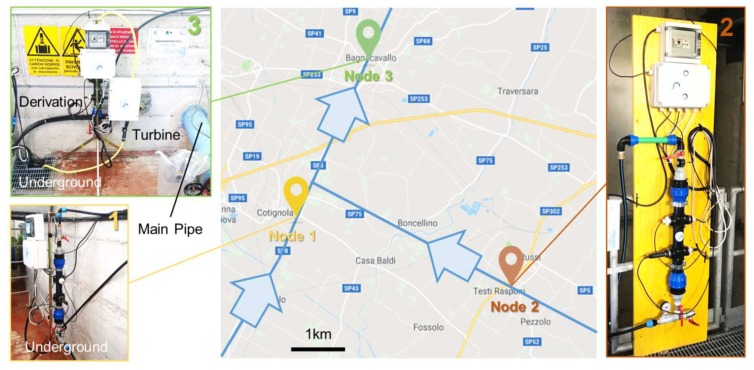
Map of the installation sites of the three nodes during the field validation in a water distribution network in Romagna, Italy. They are located in Y-junction.

The validation in the field of the proposed network took place in a drinking water distribution system in Emilia Romagna region, thanks to the support of Romagna Acque—Società delle Fonti, one of the major Italian drinking water utility, supplying water to 1.1 million citizens through a water distribution network of 604 km. Three nodes were installed to form a pilot network for demonstration purposes. Their location is shown in Figure 8: it was carefully chosen together with the network managers to be representative of a relevant case study, despite the limited number of nodes. Node 1 was installed on the pipeline transporting water from Capaccio plant, treating water from the dam of Ridracoli. Node 2 was installed on the pipeline transporting water from the Standiana plant, treating water from a surface canal connected to the river Po. Node 3 was installed on the junction line of the two pipelines, in which water was mixed and transported to the distribution network. Since the installations were conducted on branches of main pipelines characterized by consistent flow rates and high pressure (up to 12 bar), pressure regulators were interposed, setting a pressure of 2 bar and a flow rate of 5 L/min in each node. All nodes were installed inside stations (for inspection and monitoring) with ease of access. They were all powered by the power supply, and some tests of energy harvesting were performed on Node 3 (the turbine is visible in Figure 8).

Data were recorded along different time frames: here, we reported data continuously acquired for 50 days from 7 July to 26 August 2018. Due to the sensitive nature of monitoring data from field validation, the complete set of acquired data could not be reported in the present work.

**Figure 9:**
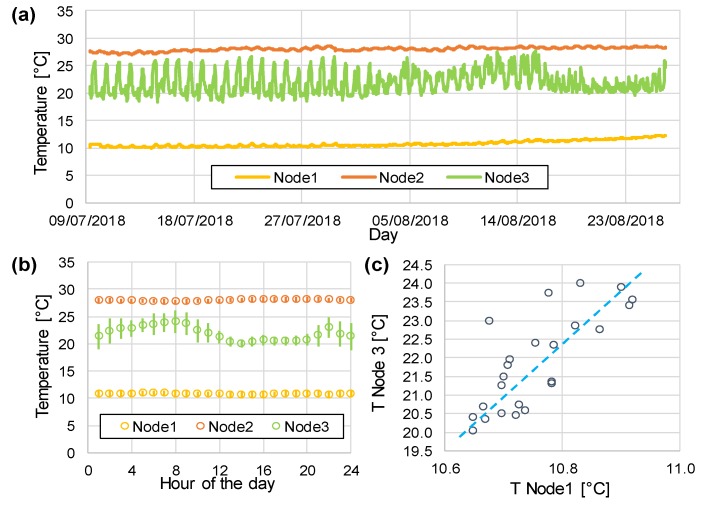
Temperature values recorded for the three nodes for 50 days (**a**), and elaboration, showing daily variations (**b**) and correlation (**c**) between Node 1 and 3.

Figure 9 reports the monitoring of water temperature variations in the three nodes. From the time tracking of Figure 9a, it is evident that the temperature in the three points was significantly different: 10 °C in Node 1, 28 °C in Node 2, and around an average of 22 °C in Node 3. The latter showed significant variations with daily periodicity, as highlighted in Figure 9b. Since the water in the pipeline monitored by Node 3 was a mixture of water from the other two pipelines, continuous modifications in the values of temperature were due to the changing ratio of blending flow rates. It was possible to observe that the temperature increased during the night. Such behavior was confirmed by the water utility staff, who reported the common practice of increasing the flow rate of water from the Standiana plant during the night to preserve water in the Ridracoli dam. Furthermore, the practice of water pumping in the basin was sometimes adopted to maximize hydroelectric energy production during the day, when electricity demand increased. A strong correlation between the temperature of input Node 1 and output Node 3 confirmed the mixing in the junction. This example demonstrated that real-time continuous monitoring of even basic parameters (and simple statistical analysis) could provide relevant information about the operation of the distribution network.

**Figure 10:**
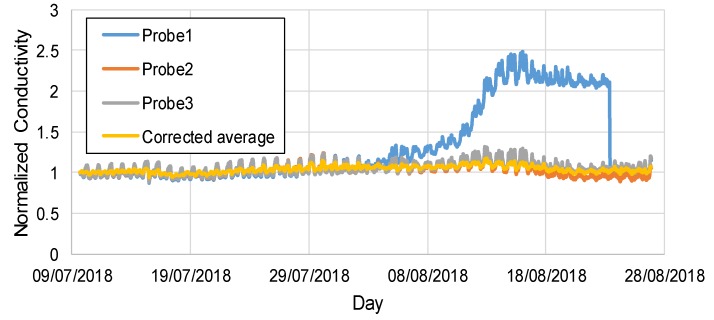
Normalized conductivity tracking for 3 identical probes in Node 3, showing fault detection and isolation of Probe 1 (whose fault was stimulated on purpose).

As an example of the validation of the self-diagnostic function of the node in the case of physically redundant sensors, three identical conductivity probes were installed in Node 3. The temporal evolution of water conductivity, normalized to the initial value for the three probes, is shown in Figure 10. It was possible to notice the strong agreement of the three couples of pins in the first part of the data series, whose averaged value was slightly higher with respect to the raw data as a consequence of the correction for temperature, also resulting in an attenuation of the characteristic daily trend. The second part of the data series was characterized by an accentuated drift in values of conductivity measured by a specific couple of pins (Probe 1). Such behavior was due to a malfunction induced on purpose, since a non-gold-coated couple of pins was used in this case, inducing the occurrence of surface modification phenomena. It was interesting to note that the algorithms automatically excluded the diverging signal and applied noise reduction functions, until performing an automatic recalibration procedure of the diverged couple of pins using the measured data from the other couples, once its signal was stabilized. No significant variations in the values of R_ion_ were observed during the field validation, indicating that water was biologically and chemically stable, and no deposits were formed.

### 5.3. Energy Harvesting

**Figure 11:**
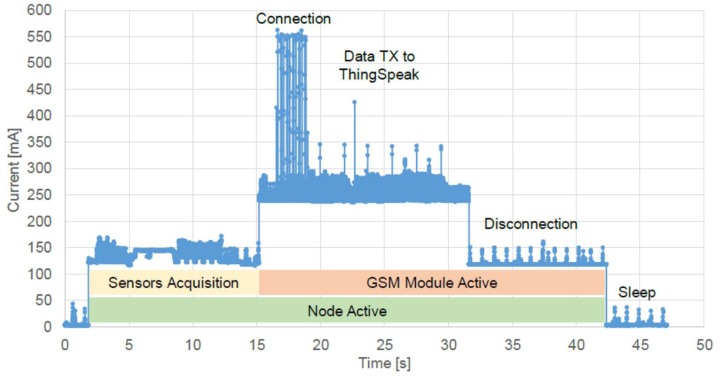
Recording of the node current consumption during an operation cycle.

One of the main differences between the final version of the electronics here presented, and previous ones [38,44] was power consumption, which has been significantly reduced (more than 30% from the initial 1.5 W). Figure 11 reports the current consumption measured with a 7-digit digital multimeter (DMM7510 by Keithley, USA, sampling at 1 MSa/s) along a full operation cycle of about 1 min. In the sleep phase, the average current consumption was around 3 mA. When the node woke up, the acquisition of sensors signals took 10 s at 120 mA. Then, the GSM modem was turned on: this was the dominant source of power dissipation. Connection to the network and transmission of data to the ThingSpeak™ server took 15 s (at 250 mA), followed by additional 10 s for disconnection (at 120 mA). If we considered a period between transmissions of 100 min, which was reasonable for monitoring these parameters, the average power consumption of the node was 42 mWh (30 mWh during the sleep mode, and 12 mWh during the active time). The very compact battery (97 × 24 × 51.5 mm^3^) inserted in the node had a capacity of 7200 mWh that would allow the operation of the node for 7 days (171.4 h).

**Figure 12:**
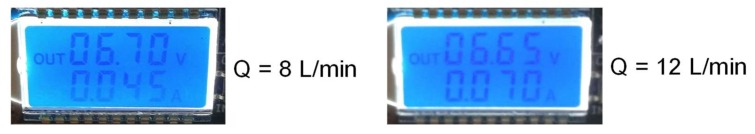
Operation of the energy harvesting unit, showing the display of the DC/DC converter for two values of the water flow rate Q in the pipe. The power manager sets an output voltage 6 V and selects the optimal battery charging current.

Electric power could be extracted from the kinetic energy of water flowing the pipe to continuously or periodically recharge the battery. A low-cost commercial device was tested to harvest energy: a Gaoxing Tech F-50 turbine (a nominal 12 V-10 W turbine). The turbine was preliminarily characterized in the laboratory: it provided a maximum current of 110 mA with a flow rate of 20 L/min. Figure 12 shows the display of the DC/DC converter with the values of its voltage and current outputs for two values of water flow rate in the node. In both cases, the power management board correctly set the output voltage to 6 V and automatically selected the proper current value to recharge the battery. The efficiency of the DC-DC converter is 90%, while the efficiency of the turbine in the conversion from kinetic to electrical power proved to be 50% for this range of flow rates. During the 100-min cycle, the battery discharged of about 1% that could be easily recovered in 1 min of water flow at rates below 10 L/min. This demonstrated the full self-powering operating mode of the sensing node.

## 6. Conclusions

We presented a multi-parameter wireless smart sensing node for real-time monitoring of water quantity and quality. The two principal features of this system are: (i) self-diagnostic capability, thanks to physical redundancy and model-based fault detection, along with a novel and quantitative slime measuring device, (ii) energetic autonomy, thanks to an efficient energy harvesting solution, applicable to compact commercial turbines. Furthermore, the absence of expensive components in the system would enable a pervasive diffusion of sensing nodes. The total cost of the node hardware, at a prototypical scale, is below 1000 €. This could be further reduced by the medium-scale manufacture of an engineered product. This cost was comparable with other standard IoT nodes for environmental monitoring. The additional hardware cost of the slime monitor is negligible, while the potential savings enabled by predictive and smart maintenance of pipelines are significant. It must be underlined that the dominant term in the total cost of each node was clearly the installation. From this point of view, in the most common case of existing infrastructures, the installation of nodes in branches already present in treatment stations and inspection wells appeared as the most straightforward solution.

Sensing performance was aligned with state of the art: conductivity resolution of 13 ppm (from 50 to 2500 µS/cm) and pH resolution of 0.01 (from 0 to 14) could be compared with 500 ppm and 0.05, respectively, demonstrated in [2]. The resolution in the estimation of the deposit thickness was about 10 µm (from 10 to 300 µm). No cross-talk among the signals at the level of the electronic board was observed. Instead, correlations among chemo-physical parameters were observed. For instance, in addition to the temperature/conductivity dependence already highlighted in paragraph 3.1, in the case of chemical contamination, both pH and conductivity levels (which are typically very stable in water distribution networks) could suddenly change. In perspective, machine learning models, leveraging the correlations among parameters, could be trained to quickly and automatically identify alterations and faults.

The results of an extensive characterization, both in the laboratory (up to 50 °C temperature, 4 bar pressure, and 1.5 m/s water velocity) and in the field with a pilot network of three nodes installed in a real water distribution network and continuously operating for 2 months, demonstrated the robustness of the proposed system. Thanks to these results, in 2019, this solution was selected as a finalist in the Zero Power Water Monitoring Horizon Prize by the European Commission.
